# New Insights into Rotavirus Entry Machinery: Stabilization of Rotavirus Spike Conformation Is Independent of Trypsin Cleavage

**DOI:** 10.1371/journal.ppat.1004157

**Published:** 2014-05-29

**Authors:** Javier M. Rodríguez, Francisco J. Chichón, Esther Martín-Forero, Fernando González-Camacho, José L. Carrascosa, José R. Castón, Daniel Luque

**Affiliations:** 1 Centro Nacional de Microbiología/ISCIII, Majadahonda, Madrid, Spain; 2 Department of Structure of Macromolecules, Centro Nacional de Biotecnología/CSIC, Cantoblanco, Madrid, Spain; 3 Instituto Madrileño de Estudios Avanzados en Nanociencia (IMDEA Nanociencia), Cantoblanco, Madrid, Spain; Institut Pasteur, France

## Abstract

The infectivity of rotavirus, the main causative agent of childhood diarrhea, is dependent on activation of the extracellular viral particles by trypsin-like proteases in the host intestinal lumen. This step entails proteolytic cleavage of the VP4 spike protein into its mature products, VP8* and VP5*. Previous cryo-electron microscopy (cryo-EM) analysis of trypsin-activated particles showed well-resolved spikes, although no density was identified for the spikes in uncleaved particles; these data suggested that trypsin activation triggers important conformational changes that give rise to the rigid, entry-competent spike. The nature of these structural changes is not well understood, due to lack of data relative to the uncleaved spike structure. Here we used cryo-EM and cryo-electron tomography (cryo-ET) to characterize the structure of the uncleaved virion in two model rotavirus strains. Cryo-EM three-dimensional reconstruction of uncleaved virions showed spikes with a structure compatible with the atomic model of the cleaved spike, and indistinguishable from that of digested particles. Cryo-ET and subvolume average, combined with classification methods, resolved the presence of non-icosahedral structures, providing a model for the complete structure of the uncleaved spike. Despite the similar rigid structure observed for uncleaved and cleaved particles, trypsin activation is necessary for successful infection. These observations suggest that the spike precursor protein must be proteolytically processed, not to achieve a rigid conformation, but to allow the conformational changes that drive virus entry.

## Author Summary

Rotavirus is responsible for more than 400,000 annual infant deaths worldwide. Its viral particle bears 60 protuberant spikes that constitute the machinery responsible for virus binding to and entry into the host cell. For efficient infection, the protein molecules that build the spike must be cleaved. Despite the importance of this activation step, the nature of the changes induced in the spike structure is unknown. According to the current hypothesis, the uncleaved spike is very flexible, and activation stabilizes the spike in an entry-competent conformation. Here we used distinct electron microscopy techniques to determine the structure of the uncleaved particle in two model rotavirus strains. Our results provide a complete structure of the uncleaved spike and demonstrate that cleaved and uncleaved spikes have similar conformations, indicating that proteolytic processing is not involved in stabilization of the spike. We suggest that spike processing is important for infection since it is necessary to allow the spike domain movements involved in rotavirus entry.

## Introduction

To initiate infection, viruses must overcome the complex membranous system that surrounds and resides within the cell. The ability of the virus to penetrate this barrier is one of the elements that define virulence and host range. Entry into the host cell is thus a key factor in viral infectivity, and a natural target for the design of efficient strategies against virus infections [1].

Rotaviruses are non-enveloped, double-stranded (ds)RNA viruses of the Reoviridae family; they infect only vertebrates, via the oral-fecal route. Their replication is generally limited to terminally differentiated enterocytes of the intestinal tract, with severe gastroenteritis restricted in the great majority of cases to the young [2]. In humans, rotavirus infection is the leading cause of medical gastroenteritis in children under five years of age [3], [4].

The rotavirus mature virion is a complex triple-layered particle (TLP) built around its inner capsid, a T = 1 icosahedral shell made of 60 asymmetric dimers of the VP2 protein [5], [6]. Inside this core, each of the eleven dsRNA segments of the viral genome is associated, below the five-fold symmetry axes, with one copy of the RNA-dependent RNA polymerase VP1, and the RNA capping enzyme VP3 [7], [8]. The inner core is surrounded by a thick shell formed by 260 trimers of the VP6 protein ordered in an icosahedral T = 13 symmetry [5], [6], [9]. This double-layered particle (DLP) constitutes the rotavirus transcriptional machinery and, characteristically of Reoviridae family members, it does not disassemble during viral infection. Rotavirus infection is effectively initiated when the DLP is released into the cytoplasm and begins synthesis of the viral transcripts.

The DLP are not infective, however, as they lack the ability to identify, bind and penetrate target cells; those functions reside in the external layer of the mature TLP [10], [11]. This external shell is formed by 260 trimers of the VP7 glycoprotein, ordered in a T = 13 icosahedral lattice. Each VP7 trimer rests on one of the VP6 trimers of the underlying DLP, anchored to small protrusions of the VP6 layer by its flexible N-terminal arm [9], [12]. Sixty spikes, formed by trimers of the VP4 protein, project from the VP7 shell. They are anchored in depressions in the VP6 layer that surround the five-fold axes, clamped by the VP7 shell that partially covers their base [6].

To become fully infectious, cell-released TLP must be digested by trypsin-like proteases from the intestinal lumen [13], [14]. This activation step cleaves the VP4 protein after three defined sites (Arg^231^, Arg^241^ and Arg^247^) to produce two main fragments, the N-terminal VP8* and the larger C-terminal VP5*. Proteolytic processing of the spikes is thought to occur through an ordered cleavage cascade that culminates in scission at Arg^247^. Cleavage after this residue is essential for membrane interactions and infectivity [15], [16].

**Figure 1:**
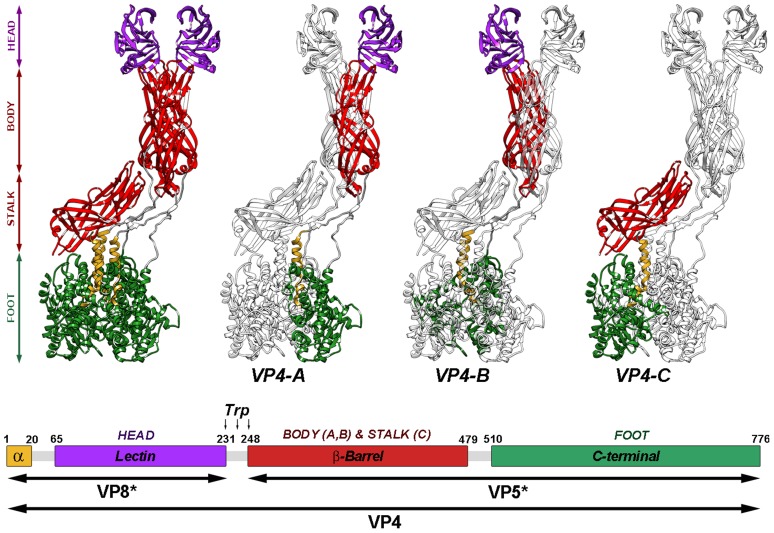
Atomic model of the VP4 spike. Ribbon representation (top) of the atomic structure of the VP4 spike (PDB entry 3IYU) color-coded to represent the spike foot (green), stalk (red), body (red) and head (purple). As indicated (VP4-A, -B and -C), each of the subunits is highlighted in color, while the remaining molecules are shown in grey. The amino- and carboxy-terminal regions of the three molecules, located contacting one another beneath the VP7 layer [6], contribute equally to the trimeric foot of the structure. The asymmetric central stalk is formed mainly by the VP5* β-barrel domain of VP4-C, which lies almost parallel to the particle surface. The distal part of the spike, constructed by equal contributions of the A and B molecules, contains the body (built by the VP5* β-barrel domains) and the globular heads (formed by VP8* lectin domains). The unusual structure of VP4-C appears to arise from asymmetries on the VP7 trimers that surround the base of the spike, which allows for distinct interactions with the base of the VP5*-C β-barrel. The globular VP8* lectin domain of the VP4-C molecule is not accounted for in the resolved structure, and is presumed to be released during proteolysis. Diagram of the organization of the VP4 chain (bottom), color-coded as in top. The VP4 proteolytic products (VP5* and VP8*) and domains are labeled. Residues delimiting domains and trypsin cleavage sites are indicated.

Analysis of the near-atomic structure of the cleaved TLP shows that the three VP4 molecules that form the spikes, termed VP4A-C [6], are organized into a complex structure that is held in place by non-covalent interactions among its components (Figure 1). Structural and biochemical data have allowed formulation of a model for rotavirus entry in which VP4 plays a role similar to that of fusion proteins during enveloped virus entry [11], [17]–[20]. Receptor binding and attachment take place through the distal lectin domains of the two VP8* molecules of chains A and B. Probably triggered by this binding, the spike components are reorganized into an extended intermediate in which hydrophobic loops of the three VP5* β-barrels, previously covered by the lectin domains (chains A and B) or by the spike body (chain C), are inserted into the target cell membrane. An additional unknown triggering event would provoke the transition from this extended intermediate to a folded-back structure, in which the hydrophobic loops now point toward the virus particle. The remarkable similarity of the initial form, the extended intermediate, and the folded-back conformation to analogous structures involved in membrane fusion of enveloped viruses suggests that the energy released by these vast conformational changes is used by rotaviruses to disrupt the cell membrane.

In this model, as with the fusion proteins of some enveloped viruses, proteolytic cleavage of the rotavirus spike protein VP4 into VP8* and VP5* primes rotavirus TLP for efficient infectivity [11], [14]. Despite the availability of the atomic structure of the trypsinized rotavirus TLP, understanding of the structural changes involved in this process has been hampered by the lack of information regarding the structure of the undigested spike. Available single particle cryo-electron microscopy (cryo-EM) reconstructions of undigested TLP show no density for VP4 projecting from the VP7 shell [21]. This suggests that proteolytic processing of VP4 triggers undetermined structural changes in the spike that result in a more stable, rigid spike structure, as described by the atomic model, that mediates rotavirus entry.

Here we used cryo-EM and cryo-ET to study the structure of the undigested rotavirus spike. Cryo-EM results showed that the structure of the uncleaved TLP of rotavirus strains SA11 and OSU is indistinguishable from that of the trypsin-digested particle, and concurs with the previously resolved near-atomic structure of the mature TLP. Cryo-ET analyses provided new insight into the organization of the uncleaved spike, and a model for its complete structure.

## Results

### Purification and characterization of TLP

**Figure 2:**
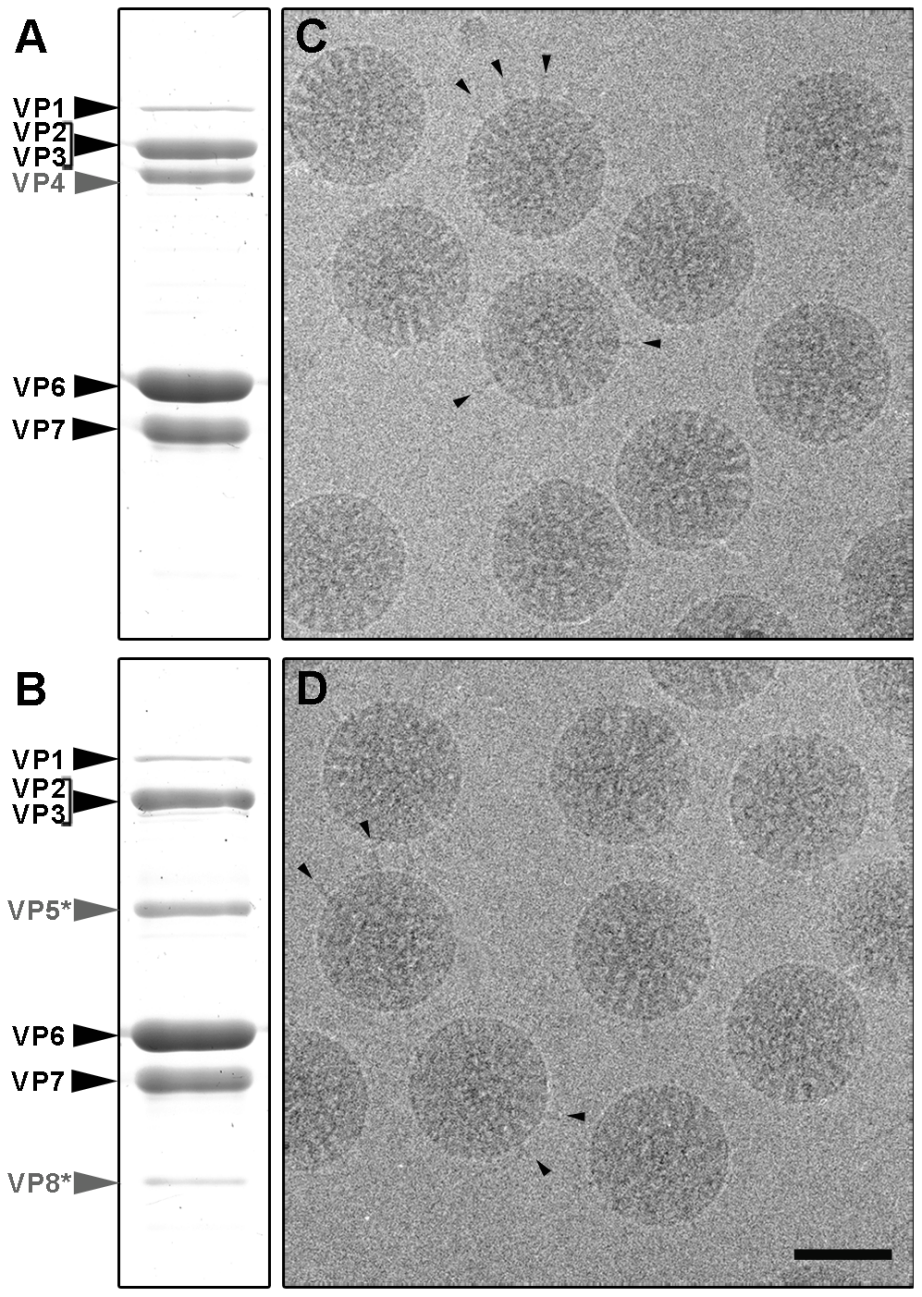
Analysis of SA11 NTR- and TR-TLP by SDS-PAGE and cryo-electron microscopy. (A, B) Coomassie blue-stained SDS-PAGE gels of purified SA11 TLP grown in the absence (A) or presence (B) of trypsin. Positions of rotavirus structural proteins (VP) are indicated. Unprocessed spike protein VP4 and its proteolytic products VP8* and VP5* are highlighted in grey. (C, D) Cryo-electron micrographs of NTR (C) and TR (D) particles. Arrowheads indicate examples of clearly defined spikes projecting from the virion surface. The bar represents 50 nm.

Rotavirus TLP were purified from cells infected with rotavirus strain SA11, in the presence of trypsin (TR-TLP), or in its absence in medium supplemented with the protease inhibitor leupeptin (NTR-TLP). Purified particles were characterized by Coomassie blue staining of SDS-PAGE gels and by western blot analysis using an antibody specific for protein VP4/VP5*. In NTR-TLP samples, the VP4 spike protein was detected mainly as the 98 kDa unprocessed precursor form (Figure 2A, S1), whereas in TR-TLP, the trypsin proteolytic products VP8* (28 kDa) and VP5* (55 kDa) account for most of the VP4 protein mass (Figure 2B, S1). During virus purification, TLP spike stability depends both on viral strain and the proteolytic state of its VP4 components [22]. To estimate the amount of spike protein preserved in our preparations, we quantified VP4 (in NTR-TLP) or its product VP5* (in TR-TLP) relative to protein VP6. Densitometric analysis of the Coomassie-stained gels produced values of 70±3% and 85±6% of the stoichiometric amount for NTR- and TR-TLP, respectively, which indicates that most spike protein was maintained to a similar extent in both samples.

Cryo-EM analysis of NTR- and TR-TLP (Figure 2C, D) showed a homogeneous population of well-preserved rotavirus particles; in some, VP4 (or VP8*/VP5*) spikes can be visualized projecting from the virion surface (Figure 2C, D, arrowheads).

### Single particle analysis of NTR- and TR-TLP

**Figure 3:**
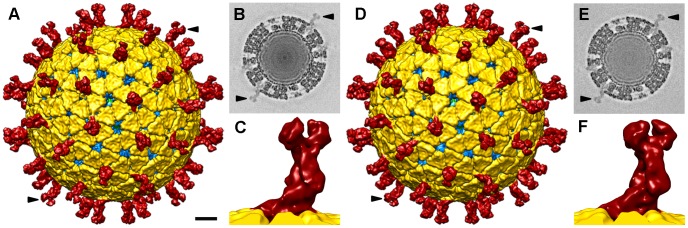
Single particle three-dimensional structures of SA11 NTR- and TR-TLP. (A, D) Surface-shaded representation of the outer surfaces of NTR (A) and TR (D) particles, viewed along an icosahedral 2-fold axis. The surfaces are radially color-coded to represent VP4 or VP8*/VP5* spikes (red), VP7 (yellow), and VP6 (blue). The density is contoured at 1σ above the mean. The bar represents 100 Å. (B, E) Transverse sections, 2.8 Å thick, taken from the maps of NTR- (B) and TR-TLP (E), parallel but displaced 34 Å from the central section, viewed along a 2-fold axis (darker, denser). Arrows indicate spikes in the surface-shaded representations in A and D and their corresponding densities in B and E. For the NTR map, the relative density of the spike contained ∼50% of the shell density; for the TR map, relative density was ∼55%. (C, F) Close up view of the NTR (C) and TR (F) spike represented as in A and D.

Three-dimensional reconstructions (3DR) were calculated for NTR- and TR-TLP with a resolution of 12.8 Å and 11.9 Å, respectively (Figure 3), obtained at a Fourier shell correlation (FSC) threshold of 0.3 (Figure S2). At the resolution achieved, the 3DR for NTR-TLP (Figure 3A–C) and TR-TLP (Figure 3D–F) were virtually indistinguishable; a difference map calculated between them showed no significant differences. The molecular architecture of the spike in both particles (Figure 3C, F) is consistent with previous structural studies for TR-TLP [6], [23], in which the cleaved spike adopts a distinctive, rigid bilobulate shape divided into a head, body, stalk and foot domains [2] (Figure 1, S3). Comparison of the relative density of spikes in both density maps using VP2-VP6-VP7 shell density as a reference (Figure 3B, E; arrowheads) showed an equivalent occupancy level (58 and 50% for NTR- and TR-TLP, respectively).

The almost identical structure observed for NTR and TR spikes (Figure 3C, F) contrasts with a previous report for the SA11-4F rotavirus strain, in which the spikes were resolved in the cryo-EM 3DR of the TR-TLP, but were undetectable for undigested TLP [21]. This difference prompted us to verify whether the presence of the protease inhibitor leupeptin or the purification method affected our results. To exclude these possibilities, NTR-TLP were produced in the absence of leupeptin, following the purification method described previously [21]. Analysis of these capsids produced a virion map equivalent to previous NTR- and TR-TLP (Figure S2 and S4). To ascertain whether these differences are strain-specific, NTR- and TR-TLP were produced from the porcine strain OSU [24] and analyzed (Figure S5, S6, S7). Cryo-EM 3DR of OSU NTR- and TR-TLP are essentially indistinguishable from each other. There were no significant differences between the OSU strain density maps or when they were compared with the 3DR of the SA11 particles.

These results show that spikes formed by trimers of undigested VP4 have a conformation similar to that of the trypsin-digested entry-primed rotavirus particles. For the simian and porcine strains analyzed in this study, ordering of the VP4 spike in a rigid bilobulate conformation is independent of trypsin digestion.

### Infectivity assays of NTR- and TR-TLP

**Figure 4:**
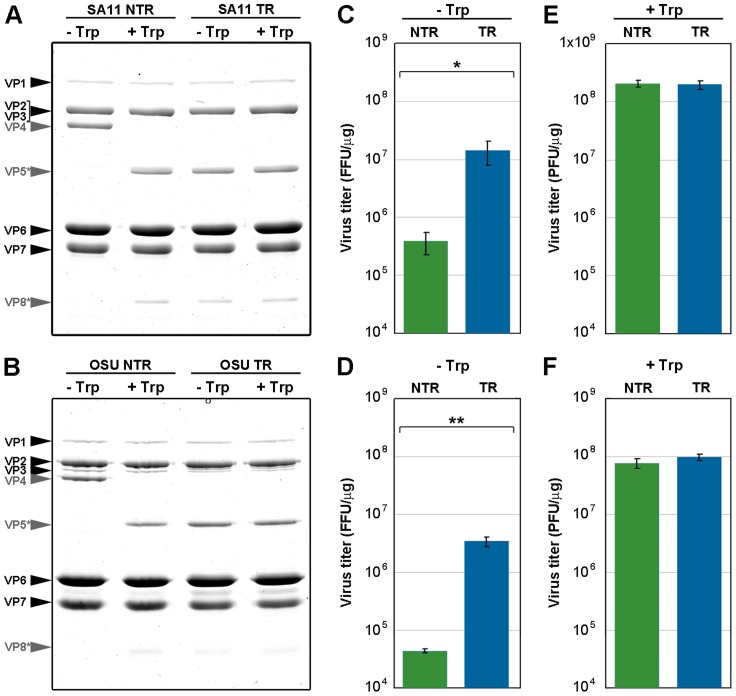
Infectivity assay of *in vitro* trypsin-treated TLP. (A, B) Coomassie blue-stained SDS-PAGE gels of purified SA11 (A) and OSU (B) TLP grown in the absence (NTR) or presence (TR) of trypsin. Samples were mock-incubated (-Trp) or incubated *in vitro* with 100 BAEE units/ml of trypsin (+Trp) (30 min, 37°C). The positions of the structural proteins (VP) are indicated. The unprocessed spike protein VP4 and its proteolytic products VP8* and VP5* are highlighted in grey. (C, D) Determination of specific infectivity of SA11 (C) and OSU (D) TLP by fluorescent focus assay in the absence of trypsin. (E, F) Determination of infectivity of SA11 (E) and OSU (F) TLP by plaque-forming assay in the presence of trypsin. Data are shown as mean ± SD. FFU, focus-forming units. PFU, plaque-forming units. * p<0.02, ** p<0.005.

To study the effect of trypsin cleavage on infectivity, we determined the specific infectivity of both TLP types, before and after trypsin treatment (Figure 4). *In vitro* trypsin treatment of NTR-TLP resulted in disappearance of the VP4 band and appearance of proteolytic products VP8* and VP5*; there were no detectable changes in the protein profile following similar treatment of TR-TLP (Figure 4A, B, grey arrows). Due to the trypsin dependence for rotavirus plaque formation, the specific infectivity of mock-treated TLP was evaluated in a focus-forming assay in the absence of trypsin, in which expression of the viral protein NSP4 was used to detect infected cells (Figure 4C, D). The background infectivity in NTR-TLP preparations is attributed mainly to the activity of proteases released during cell lysis [14]. Undigested NTR-TLP had ∼1.5 logarithmic units lower specific infectivity than undigested TR-TLP (1.89 for SA11, p<0.02; 1.56 for OSU, p<0.005). The specific infectivity of trypsin-treated TLP was determined in a plaque-forming assay that directly tests for infective particles able produce infective progeny. In this assay, trypsin-activated NTR- and TR-TLP showed specific infectivity levels with no significant differences (Figure 4E, F). The results demonstrate that trypsin treatment enhances NTR-TLP specific infectivity to a level similar to that of trypsin-activated TR-TLP, as shown for several rotavirus strains [14], [22]. Although we detected no structural differences between NTR- and TR-TLP by single particle cryo-EM 3DR, proteolytic processing of VP4 is necessary for efficient viral infectivity.

### Cryo-electron tomography of NTR- and TR-TLP

**Figure 5:**
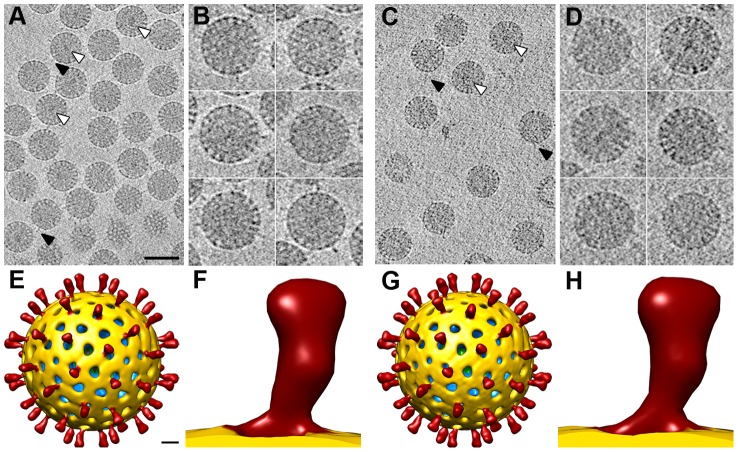
3DR of SA11 TLP from cryo-electron tomography. (A, C) Slice through the xy plane of the reconstructed cryo-electron tomograms of NTR- (A) and TR-TLP (C). The bar represents 100 nm. White arrowheads indicate examples of electrodense structures inside particles. Black arrowheads indicate examples of spikes on the outer particle surface of the virions. (B, D) Gallery of central slices through extracted NTR- (B) and TR-TLP (D). (E, G) Surface-rendered model of the averaged NTR- (E) and TR-TLP (G) calculated from the extracted subtomograms and viewed along an icosahedral 2-fold axis. The bar represents 100 Å. (F, H) Close up view of the NTR (C) and TR (F) spike represented as in E and G.

The densities for the spikes in single particle cryo-EM analysis of NTR- and TR-TLP can be assigned to most of the three VP4 polypeptide chains (A, B and C) that compose the spikes, with the exception of the lectin domain of the VP4-C molecule (Figure 1). It is suggested that this lectin domain is lost during TR-TLP preparation [6]. In NTR spikes, it is covalently bound to the rest of the VP4 molecule, which indicates that the density for this domain is smeared due to the icosahedral averaging imposed during 3DR. We used cryo-ET to overcome this limitation and study the structure of SA11 strain NTR-TLP, a strategy used successfully to analyze surface proteins of enveloped viruses and non-symmetric elements in icosahedral viruses [25], [26]. We collected several cryo-tomographic series of projection images of SA11 NTR- and TR-TLP covering the angular range from −66° to +66°. Tomograms from these tilt-series were reconstructed to obtain the averaged map of 607 and 242 single NTR and TR particles, respectively (Figure 5A–D).

Virtual sections from individual particles showed an electron-dense region near the geometric center of some particles (∼30% and ∼15% of NTR and TR, respectively, Figure 5A, C, white arrowheads), which can only be associated with the genomic dsRNA. This structure is reminiscent of the dsRNA condensation detected by cryo-EM analysis of TR-TLP in the presence of ammonium ions at high pH [27], although the biological importance of this feature is not clear. Projecting densities that correspond to the virion spikes were clearly visible on the outer particle surface of both samples (Figure 5A, C, black arrowheads).

### Subtomogram averaging

Subtomograms of individual virions were extracted (Figure 5B, D), aligned and averaged, considering icosahedral symmetry to optimize determination of the origin and orientation of each virion subvolume. In accordance with our results in single particle cryo-EM 3DR (Figure 3), the spikes in the final averaged density maps of the NTR- and TR-TLP showed no significant differences (Figure 5).

**Figure 6:**
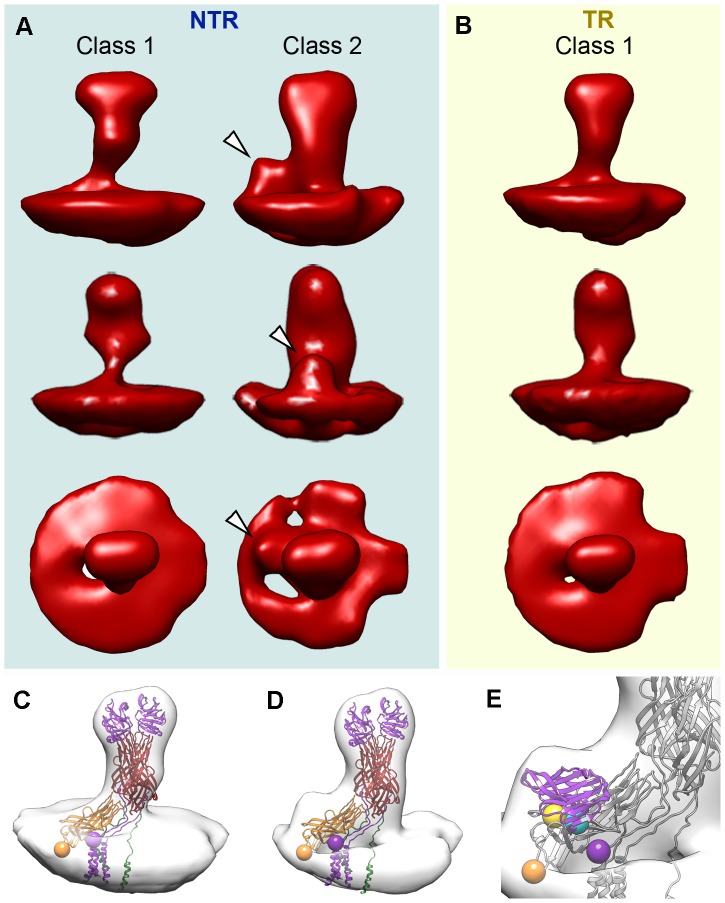
Tomogram averaging and classification of NTR and TR spikes. (A, B) Surface-rendered model of averaged tomograms, reference-free classified, for NTR (A) and TR (B) spikes. Top and middle rows show two side views of the averages related by a 90 degree rotation. The bottom row shows the top view of the averages. Arrowheads indicate an extra density at the base of the spike stalk in the class 2 NTR average, which is absent in NTR and TR class 1 averages. (C) Surface-rendered model of the class 1 TR spike fitted with the VP4 atomic model (PDB entry 3IYU). VP8* molecules A and B are in purple, VP5* molecules A and B are in red, and VP5* C is in orange. The last resolved residue of VP8* (Lys^29^, purple) and the first resolved residue of VP5* (Glu^264^, orange) are indicated by spheres for the VP4-C molecule. (D) Surface-rendered model of the class 2 NTR spike fitted with the VP4 atomic model and represented as in C. (E) Close up view of the NTR spike fitted with a single VP8* lectin domain superimposed on the extra density detected at the base of the stalk. First (Leu^65^, cyan) and last (Leu^224^, yellow) residues for the fitted domain are represented as spheres (arrowheads).

This alignment process not only generated an average structure for the virions, but also allowed determination of the origin and orientation of each particle subvolume relative to the average density. We used this information, combined with knowledge of each spike position in the average structure and of their icosahedral symmetry relationships, to computationally extract and orient 36,420 and 14,580 spike subtomograms from the original non-symmetrized densities of the NTR- and TR-TLP, respectively. The extracted spike subtomograms were then reference-free classified using a maximum-likelihood algorithm that takes into account the missing wedge information [28]. In this process, no spike density was detected projecting from the VP7 shell for 28% of the NTR and 40% of the TR spike subtomograms, which could correspond to positions where spikes have been lost and those in which they are flexible or disordered. For the remaining subtomograms, the classification process converged to two classes for the subtomograms of the NTR spikes, whereas there was only one class in the TR spike subtomograms (Figure 6A, B). Positions in which no density was detected and those assigned to a class were distributed randomly at the virus particle surface.

Class 1 contains the majority of the NTR and TR spike subtomograms (47% for NTR, 60% for TR) and yielded averages that greatly resemble the structure of the NTR- and TR-TLP spikes obtained when icosahedral symmetry was imposed on the 3DR from cryo-EM (Figure 3) or cryo-ET data (Figure 5). Fitting of the atomic coordinates of trypsinized VP4 [6] into class 1 averages showed good agreement for the spike stalk, body and head (Figure 6C). The lectin domain of the VP4-C molecule is the only VP4 region not accounted for by either the atomic model or the density maps. Although for the TR spike it could be argued that this lectin domain is lost after proteolytic cleavage, the existence of this class for the NTR subtomograms, in which the lectin domain is covalently bound to the remainder of the VP4-C molecule, indicates that this domain is more flexible in this molecule than the equivalent domain of the other VP4 molecules. Class 2 was detected only for NTR spikes, and contained 25% of the subtomograms. The averaged spike density is similar to that of class 1, with an additional bulge at the base of the spike stalk (Figure 6A, arrowheads). When the atomic coordinates of the TR VP4 (Figure 6D) were fitted to this averaged volume, the unassigned bulge density was compatible in size and shape with a single lectin domain (Figure 6E). The terminal residues of this domain (Leu^65^ and Leu^224^, cyan and yellow spheres, respectively, in Figure 6E) were located just above the last defined residues in the atomic model for the VP4-C flanking regions (Lys^29^ and Glu^264^, purple and orange spheres, respectively, in Figure 6C–E) [6]. The evidence suggests that this density corresponds to the lectin domain of the VP4-C molecule that contributes to the spike stalk, and that the average density for class 2 subtomograms reflects the complete structure of the undigested rotavirus spike.

## Discussion

Recent reports suggest that the conformational changes undergone by the rotavirus spike protein VP4 are the main driving force behind membrane disruption and virus entry into the host cell [11], [17], [20]. For this process to be efficient, the rotavirus particle must be primed by proteolytic cleavage of VP4 into its mature products, VP8* and VP5*. Despite the availability of an atomic structure for the trypsinized rotavirus particle, our understanding of the molecular mechanisms that underlie the proteolytic enhancement of rotavirus infectivity has been hindered by the lack of a structure of the undigested spike. The aim of this study was to determine, using cryo-EM and cryo-ET, the structure of the uncleaved rotavirus to improve comprehension of the structural mechanisms underlying the proteolytic enhancement of rotavirus infectivity.

Single particle cryo-EM reconstructions of the NTR- and TR-TLP of the simian SA11 and porcine OSU rotavirus strains yielded structures that showed no differences among them and were consistent with the near-atomic structure of the rotavirus RRV strain TR-TLP [6]. This indicates that the overall spike conformation achieved by the three complete undigested VP4 molecules is essentially maintained after trypsin activation. The interactions of the unprocessed VP4 molecules with the VP6 and VP7 trimers during assembly in the six-coordinated cavity are thus sufficient to configure these molecules in the A, B or C conformations, according to their relative position. This structure for the undigested spike is consistent with the available structural data for the TR-TLP particle, since the length of the trypsin-released segment of the loops is sufficient to bridge the observed C and N termini of VP8* and VP5*, respectively [6]. This architecture would also explain the differential trypsin sensitivity of unassembled and spike-assembled VP4 [29], as most putative trypsin-sensitive sites are protected in the latter, with the exception of the known cleavage sites Arg^230^, Arg^241^, Arg^247^ and the proposed cleavage site at Lys^29^ in VP8* of VP4-C [6]. The observation of an equivalent structure at the cell interaction domain of cleaved and uncleaved spikes is also consistent with the finding that both particle types bind similarly to cell membranes [30].

Although trypsin cleavage has only a limited effect on spike structure, infectivity experiments showed that protease cleavage of the spike protein is essential for high levels of specific viral infectivity. These data suggest that whereas the fold shared by NTR and TR particles is attachment-competent, the spike must be primed by cleavage for further conformational changes. In this dependence on proteolytic activation, as well as in the nature of the conformational changes during viral entry, VP4 has a striking similarity to some membrane fusion proteins of enveloped viruses [11], [17]. The class 1 viral fusion glycoproteins are synthesized as precursors that require protease activation for virus infectivity [31], [32]. This necessary activation step is associated with relatively little structural rearrangement of the fusion protein, similar to our observations for rotavirus. For example, the atomic structures of the uncleaved and cleaved forms of the influenza prefusion hemagglutinin protein are largely superimposable, except for the 19-residue segment that includes the protease cleavage site [33], [34]. In the uncleaved form, these residues are in an exposed loop; after cleavage, the fusion peptide in the newly-formed N terminus is buried in a nearby hydrophobic cavity. In the paramyxovirus parainfluenza virus 5, structural movements between the cleaved and uncleaved forms of the fusion protein are also limited to the residues that compose and surround the protease recognition site; since the residues that compose the hydrophobic fusion peptide are already packed in the uncleaved structure, the conformational changes are even more subtle [35].

The cryo-EM-derived atomic structure of the rotavirus TR-TLP does not account for the lectin domain of VP4-C [6]. Results were similar for the NTR- and TR-TLP 3DR of both strains used in this study. The absence of density for this domain, which in NTR-TLP is covalently bound to the rest of the VP4-C subunit, indicates greater flexibility of this part of the molecule, resulting in loss of the density due to the icosahedral averaging imposed during 3DR. The classification of cryo-ET subvolumes allowed us to overcome this limitation, and the averages for the distinct classes provides a deeper understanding of the spike structure.

**Figure 7:**
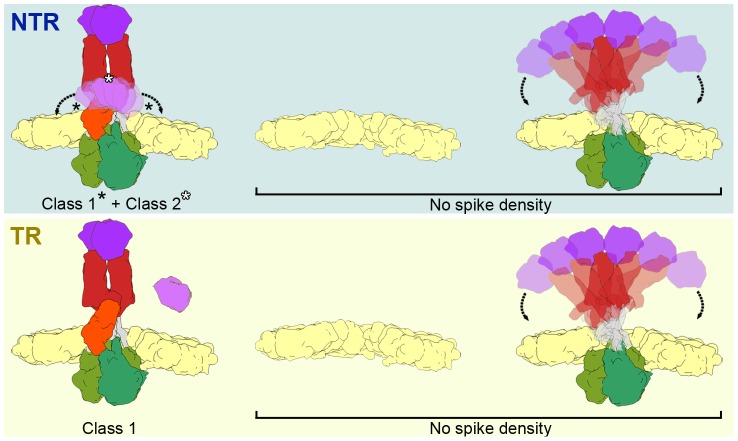
Model of the conformational states of the rotavirus spike in NTR- and TR-TLP. NTR spikes have a flexible VP4-C lectin domain at the base of the stalk (top, left). NTR Class 1 represents the average of more distal positions (black asterisks) and class 2 is generated by the average of more central positions (white asterisk) of the flexible lectin domain. The proteolytic processing of spike components releases the VP4-C lectin domain (if an additional cleavage at Lys^29^ is produced) or increases its flexibility in TR class 1 spikes (bottom, left). The fraction of NTR (top, right) and TR (bottom, left) subvolumes in which no spike density is detected could correspond to a mixture of positions without spikes (or with damaged spikes) and with highly flexible spikes.

The atomic model of the TR-TLP spike was fitted with good agreement to the averaged density of class 1, the most abundant class for NTR-TLP spikes and the only class detected in TR-TLP spikes. Neither the averaged maps for class 1 nor the atomic coordinates detect the VP4-C lectin domain. This domain is also absent in all 3DR derived from cryo-EM single particle analysis, as well as the in the icosahedrally averaged maps of virion volumes obtained by cryo-ET. In the case of NTR-TLP, this domain is covalently linked to the remainder of the VP4-C molecule; classes 1 and 2 thus contain three complete VP4 molecules in NTR samples. Nonetheless, we only identified a density compatible with this domain in the class 2 averaged maps, observed only in NTR-TLP subtomograms. This apparent contradiction could be a result of VP4-C lectin domain flexibility. As illustrated in Figure 7, spike subtomograms in which this domain is located near a central position (25%, white asterisk in Figure 7, top) generate the class 2 averaged density. Class 1 subvolumes (47%) are composed of densities in which the lectin domain swings away from this central position (black asterisks in Figure 7, top) and whose averaging results in disappearance of the density. In the case of the spike subtomograms from TR-TLP, the lack of a class equivalent to class 2 might indicate that trypsin digestion further increases lectin domain flexibility or, as suggested by analysis of the TR-TLP atomic structure, that a second cleavage in Lys^29^ releases the domain from the spike (Figure 7, bottom) [6].

In both samples, a fraction of the subtomograms (28% of NTR and 40% of TR) rendered averaged maps that show no density in the regions in which the spikes are located (Figure 7, No Spike density). These subtomograms would include positions in which the spike is very flexible, has been lost, or its structure has been damaged due to the purification conditions (particularly the organic extraction procedure [22]). Biochemical analysis of the TLP preparations (Figure 2) shows that a percentage of the predicted VP4 molecules is not present in the purified NTR (30%) or TR (15%) particles. Although we cannot rule out the possibility that all volumes in these fractions correspond to positions at which the spike has been lost, the large percentage of subtomograms included in this group, especially in the case of TR-TLP positions, suggests that they also include positions in which the spike is present but is highly flexible and whose density is lost after averaging (Figure 7). It can also be argued that the greater percentage of tomograms in this fraction in TR-TLP reflects a greater degree of freedom induced by trypsin cleavage of VP4 into its products, VP8* and VP5*. The redundancy derived from the virion icosahedral symmetry could explain its tolerance for the presence of defective spikes in the particle. Indeed, the relatively low occupancy observed in *in vitro* recoated particles is sufficient for a very efficient infectivity [36].

Previous cryo-EM single particle analysis of the NTR-TLP structure did not detect a density corresponding to the spike structure [21]. These different results could be attributed to the nature of the SA11-4F strain used in the previous 3DR, which is unique in its ability to produce small clear plaques in the absence of trypsin, and for its extreme sensitivity to protease digestion [37]. These characteristics arose from the reassortment of a segment 4 that encodes a VP4 gene from a bovine rotavirus on a SA11 genetic background [38], [39]. The molecular basis of the distinct behavior of SA11 and SA11-4F strains is thus probably due to changes in the sequence of the reassorted VP4 molecule (129 changes in a total of 776 residues). The large number of variations makes it impossible to determine the amino acids involved. Nonetheless, the VP4 region that interacts with VP6, which mediates spike foot recruitment and trimerization, is conserved. There are several changes in the VP7-contacting region of VP4, which dictates how the spike projects, as well as in the preceding linker, which is not resolved in the atomic structure [6]. The SA11 and OSU strains used in this study show more standard behavior, with no evidence of interspecies reassortment. Based on our results, we hypothesize that whereas most NTR-TLP spikes in SA11 and OSU strains are stabilized, the majority of VP4 molecules in the SA11-4F strain are not stabilized, and the spike density is lost when it is averaged and icosahedral symmetry is imposed. We suggest that the structures we observed for SA11 and OSU uncleaved spikes represent a more general architecture, and that SA11-4F is an extreme example of uncleaved spike flexibility.

In summary, our cryo-EM studies of two unrelated model rotavirus strains show that in the absence of trypsin cleavage, most of the three VP4 molecules that compose the spike adopt a stable conformation, similar to that of the mature cleaved TLP. Cryo-ET results reinforce this conclusion and, additionally, evidence the great flexibility of the lectin domain of the VP4-C chain, providing us with a model for the complete structure of the uncleaved spike. Our findings indicate that cleavage of the spike proteins is important for infectivity because it influences later events, probably those conformational changes proposed to mediate membrane disruption.

## Materials and Methods

### Virus production, purification and titration

The monkey epithelial cell line MA104 (ECACC 85102918) was cultured in MEM with 10% fetal calf serum and used between passages 7 and 24. The simian agent 11 rotavirus strain (SA11, [39], [40]) was obtained from Dr. J. Buesa, (University of Valencia, Valencia, Spain), and the Ohio State University porcine strain (OSU; [24]) from Dr. O. Burrone (ICGEB, Trieste, Italy). Both strains were cloned by four successive plaque isolation steps in MA104 cells. The clones SA11-C4111 and OSU-C5111 were selected and amplified. cDNAs of the complete genome segments of the two viral clones were obtained following procedures described by Potgieter et al. [41], cloned in the plasmid pJET1.2 (Fermentas) and sequenced. Given the complex history of the SA11 family of viruses [39], [42], we analyzed the genome sequence of the SA11-C4111 strain. The results showed a SA11-like genome very similar to that of the N5 strain [43], with no evidence of genomic reassortment. The amplified viruses were used within three passages of the last plaque isolation step. For simplicity, here we refer to these clones as SA11 and OSU.

TLP were produced and purified by ultracentrifugation in CsCl gradients as described by Patton et al. [44], with minor modifications (see supporting Text S1). In parallel experiments, aliquots of the TLP samples were vitrified (below), treated with SDS-PAGE sample buffer for biochemical characterization or analyzed for infectivity. Viral infectivity was determined by plaque assays and fluorescent focus assays, essentially as described by Arnold et al. [45] with minor modifications (see supporting Text S1).

### Cryo-electron microscopy

NTR and TR-TLP samples (5 µl) were applied to one side of acetone-washed Quantifoil R 2/2 holey grids, blotted, and plunged into liquid ethane using a Leica EM CPC cryo-fixation unit. Cryo-EM images were recorded in low-dose conditions (∼10 e^−^/Å^2^), in a Tecnai G2 electron microscope operating at 200 kV. For SA11 NTR and TR samples, micrographs were recorded at a nominal magnification of 50,000X. SA11 NTR-TLP produced in the absence of leupeptin and OSU samples were imaged on a FEI Eagle 4k CCD at a detector magnification of 67,873X (2.21 Å/pixel sampling rate).

### Image processing

General image processing operations were performed using Bsoft [46] and Xmipp [47] software packages. Graphic representations were produced by UCSF Chimera [48]. Micrographs were digitized using a Nikon Super CoolScan 9000 ED at a 6.35 µm step size, or a Zeiss TD scanner at a 7 µm step size, to yield 1.27 Å or 1.4 Å pixel size in the specimen, respectively. X3d [49] was used to manually select 3,802, 2,980, 2,150, 4,100 and 2,100 individual images for SA11 NTR-, SA11 TR-, SA11 NTR - leupeptin, OSU NTR- and OSU TR-TLP, respectively. Defocus was double determined with bshow [46] and CTFfind [50], and the CTF were corrected in the images by flipping phases in the required lobes. The published structure of the rotavirus VP7-recoated particle [12], low-pass filtered to 30 Å, was used for the initial determination of the origin and orientation of each particle for all samples. As these recoated particles lack VP4, any model bias at the spike density is avoided. Xmipp iterative projection matching was carried out to determine and refine the origin and orientation of each particle. Reconstructions were computed using interpolation in Fourier space. After each iteration, resolution was assessed by FSC, applying a correlation limit of 0.5 between two independent reconstructions. The final reconstructions combined 3,421 images for SA11 NTR, 2,682 for SA11 TR, 1,935 for SA11 NTR - leupeptin, 3,690 for OSU NTR and 1,890 for OSU TR. Amplitude decay was corrected by adjusting the spatial frequency components of the cryo-EM maps to the decay profile of the atomic map of rotavirus TLP (PDBs 3N09 and 2GH8). This adjustment was applied in the frequency range from 340 Å to the maximum resolution achieved, and a soft low-pass filter was applied. Amplitude decay was also calculated and corrected with Embfactor [51], with similar results.

### Cryo-electron tomography

Samples were mixed with 10 nm gold particles and vitrified as described above. Tomographic tilt-series were recorded in a Tecnai G2 electron microscope operating at 200 kV on a FEI Eagle 4k CCD using the FEI Xplore3D software at a detector magnification of 32,609X (4.6 Å/pixel sampling rate) every 1.5 degrees. Images were acquired with a defocus ranging from 5 to 8 µm, and an accumulated total dose from 90 to 120 e/Å. Tilted series were processed using IMOD [52] and CTF corrected using TOMOCTF [53]. A final number of 4 and 3 tomograms were reconstructed for SA11 NTR and TR samples, respectively, using unfiltered weighted back projection algorithms implemented in the TOMO3D package [54] to recover all the high frequencies for subvolume averaging process.

### Subtomogram averaging and classification

IMOD software was used to manually select, and extract 607 and 243 virions from SA11 NTR and TR samples, respectively. Subvolumes were aligned and averaged using the MLTomo routine [28] from Xmipp, considering icosahedral symmetry to optimize the origin and orientation determination at this step. This process was performed using the previously obtained cryo-EM 3DR as initial template, as well as in a reference-free manner. Both approaches yielded equivalent results. The final averaged volumes were used to determine the 3D position of the spikes. This information, together with the origin and orientation assigned to each particle, were used to automatically extract 36,420 and 14,520 subvolumes for each spike in the original asymmetric tomograms of SA11 NTR and TR, respectively. The spike subtomograms were classified and averaged with MLTomo. Independently of the initial number of classes used, the classification of the NTR spikes converged to 3 groups containing 10,084 (28%, no spike density), 17,281 (47%, class 1) and 9,055 (25%, class 2) volumes, whereas classification of the SA11 TR spikes converged to two groups with 5,810 (40%, no spike density) and 8,710 (60%, class 1) volumes.

### Accession codes

Sequences corresponding to the different SA-C4111 and OSU-C5111 genomic segments are deposited in GenBank (accession numbers in Table S1). The 3DR and averaged subtomograms are deposited in the Electron Microscopy Data Bank (EMDB; accession codes in Table S2).

## Supporting Information

Figure S1 **Western blot analysis of SA11 and OSU NTR- and TR-TLP.** (A) Coomassie blue-stained SDS-PAGE gel of purified SA11 and OSU NTR- and TR-TLP. Positions of structural viral proteins (VP) are indicated. Position of unprocessed spike protein VP4, and its products VP5* and VP8* are highlighted (grey). (B) Western blot analysis. A gel similar to that in A was immunoblotted with an anti-VP4 antibody that recognizes the precursor VP4 and its product VP5*.
(TIF)

Figure S2 **Assessment of the resolution cryo-EM 3DR of SA11 NTR and TR TLP.** (A) FSC resolution curves were calculated for SA11 NTR-TLP (blue, continuous line), NTR-TLP grown in absence of leupeptin (purple, dashed line) and TR-TLP (blue, dashed line). For the 0.5 threshold the values for SA11 NTR-, TR- and NTR-TLP without leupeptin were 15.4, 14.5 and 15.9 Å, respectively; values for the 0.3 threshold were 12.8, 11.9 and 13.3 Å, respectively.
(TIF)

Figure S3 **Fit of the atomic coordinates into the cryo-EM map.** (A) Scheme of the NTR spike and its interaction with VP6 and VP7 shells. Proteins and color coding are indicated. (B) Superposition of the atomic coordinates of RRV TR-TLP (PDBs 3N09 and 2GH8) [6] and the SA11 NTR-TLP cryo-EM map. Coordinates and densities are color-coded as in A.
(TIF)

Figure S4 **Biochemical and structural analysis of SA11 NTR-TLP grown in the absence of leupeptin.** (A) Coomassie blue-stained SDS-PAGE gels of purified SA11 TLP grown in the absence of trypsin and leupeptin. Positions of structural viral proteins (VP) are indicated. Position of unprocessed spike protein VP4 is highlighted in grey. (B) Cryo-electron micrograph of vitrified particles. The bar represents 50 nm. (C) Surface-shaded representation of the outer surface of the 3DR, viewed along an icosahedral 2-fold axis. The surface is radially color-coded to represent VP4 (red), VP7 (yellow) and VP6 (blue). The density is contoured at 1 σ above the mean. (D) Transverse sections, 2.8 Å thick, taken from the maps parallel but displaced 34 Å from the central section, viewed along a 2-fold axis (darker, denser). Arrows indicate spikes in the surface-shaded representations in C and their corresponding densities in D. (E) Close-up view of the spike represented as in C.
(TIF)

Figure S5 **Analysis of OSU NTR- and TR-TLP by SDS-PAGE and cryo-EM.** (A, C) Coomassie blue-stained SDS-PAGE gels of purified OSU TLP grown in the absence (A) or presence (C) of trypsin. Positions of structural viral proteins (VP) are indicated. Unprocessed spike protein VP4 and its proteolytic products VP8* and VP5* are highlighted in grey. (B, D) Cryo-electron micrographs of NTR (B) and TR (D) particles. The bar represents 50 nm.
(TIF)

Figure S6 **Assessment of the resolution cryo-EM 3DR of OSU NTR and TR TLP.** FSC resolution curves were calculated for OSU NTR-TLP (red, continuous line) and TR-TLP (red, dashed line). For the 0.5 threshold, values for OSU NTR- and TR-TLP were 16.2 and 17.4 Å, respectively; values for the 0.3 threshold were 14.3 and 15.4 Å, respectively.
(TIF)

Figure S7 **Single-particle three-dimensional structures of OSU NTR- and TR-TLP.** (A, D) Surface-shaded representation of the outer surfaces of NTR (A) and TR (D) particles, viewed along an icosahedral 2-fold axis. The surfaces are radially color-coded to represent VP4 or VP8*/VP5* spikes (red), VP7 (yellow) and VP6 (blue). The density is contoured at 1σ above the mean. The bar represents 100 Å. (B, E) Transverse sections, 2.8 Å thick, taken from the maps of NTR (B) and TR (E) TLP, parallel but displaced 34 Å from the central section, viewed along a 2-fold axis (darker, denser). Arrows indicate spikes in the surface-shaded representations in C and D and their corresponding densities in B and E. (C, F) Close up view of the NTR (C) and TR (F) spike represented as in A and D.
(TIF)

Table S1 **GenBank accession numbers for SA-C4111 and OSU-C5111 genomic segments.**
(DOC)

Table S2 **EMDB accession codes for density maps.**
(DOC)

Text S1 **Supporting Materials and Methods.**
(DOC)
