# Supplementary figures and images for: New Insights into Rotavirus Entry Machinery: Stabilization of Rotavirus Spike Conformation Is Independent of Trypsin Cleavage
Source: PLoS Pathog. 2014 May 29;10(5):e1004157. doi: 10.1371/journal.ppat.1004157 (PMC4038622; doi:10.1371/journal.ppat.1004157)

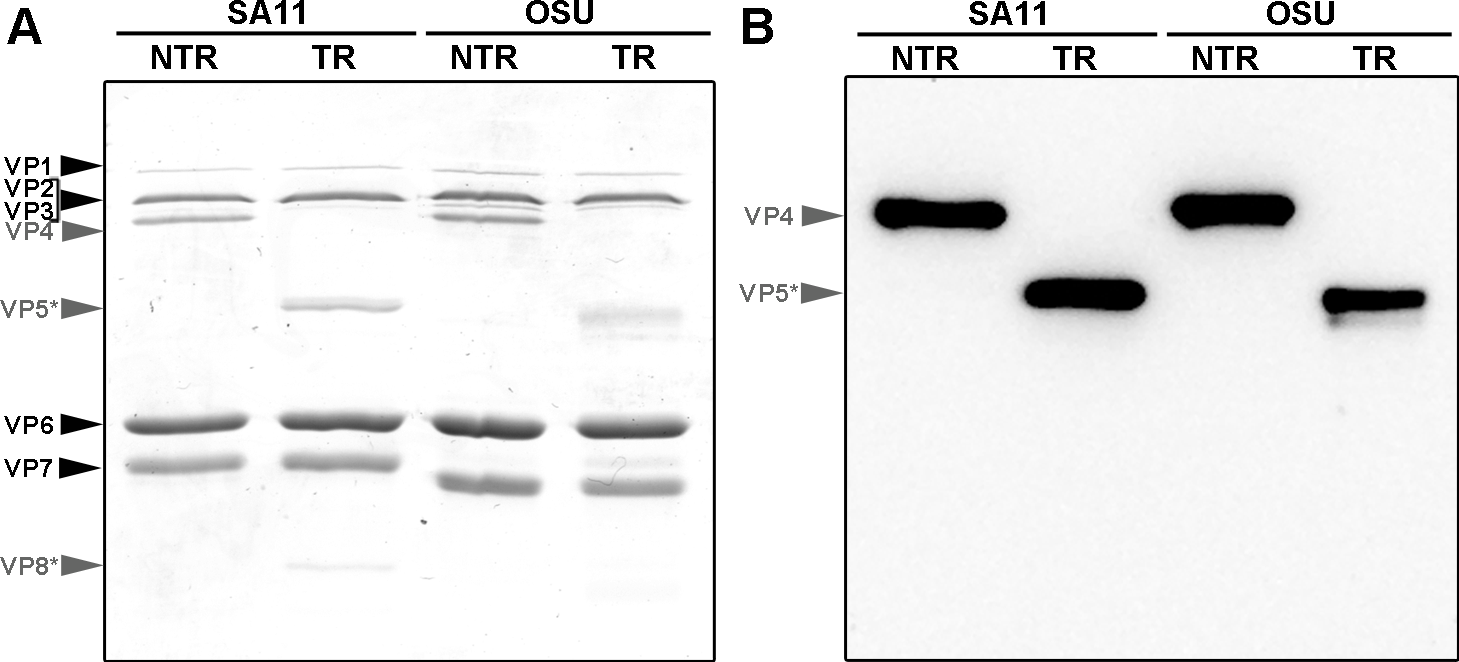

Supplement: Figure S1 — Western blot analysis of SA11 and OSU NTR- and TR-TLP. (A) Coomassie blue-stained SDS-PAGE gel of purified SA11 and OSU NTR- and TR-TLP. Positions of structural viral proteins (VP) are indicated. Position of unprocessed spike protein VP4, and its products VP5* and VP8* are highlighted (grey). (B) Western blot analysis. A gel similar to that in A was immunoblotted with an anti-VP4 antibody that recognizes the precursor VP4 and its product VP5*. (TIF) [file ppat.1004157.s001.tif]

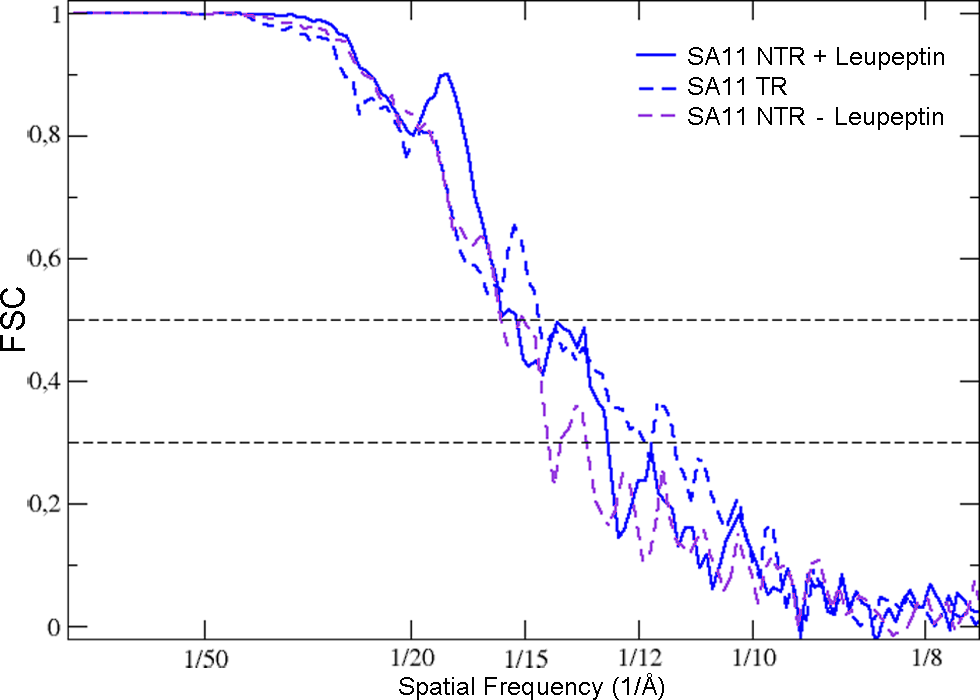

Supplement: Figure S2 — Assessment of the resolution cryo-EM 3DR of SA11 NTR and TR TLP. (A) FSC resolution curves were calculated for SA11 NTR-TLP (blue, continuous line), NTR-TLP grown in absence of leupeptin (purple, dashed line) and TR-TLP (blue, dashed line). For the 0.5 threshold the values for SA11 NTR-, TR- and NTR-TLP without leupeptin were 15.4, 14.5 and 15.9 Å, respectively; values for the 0.3 threshold were 12.8, 11.9 and 13.3 Å, respectively. (TIF) [file ppat.1004157.s002.tif]

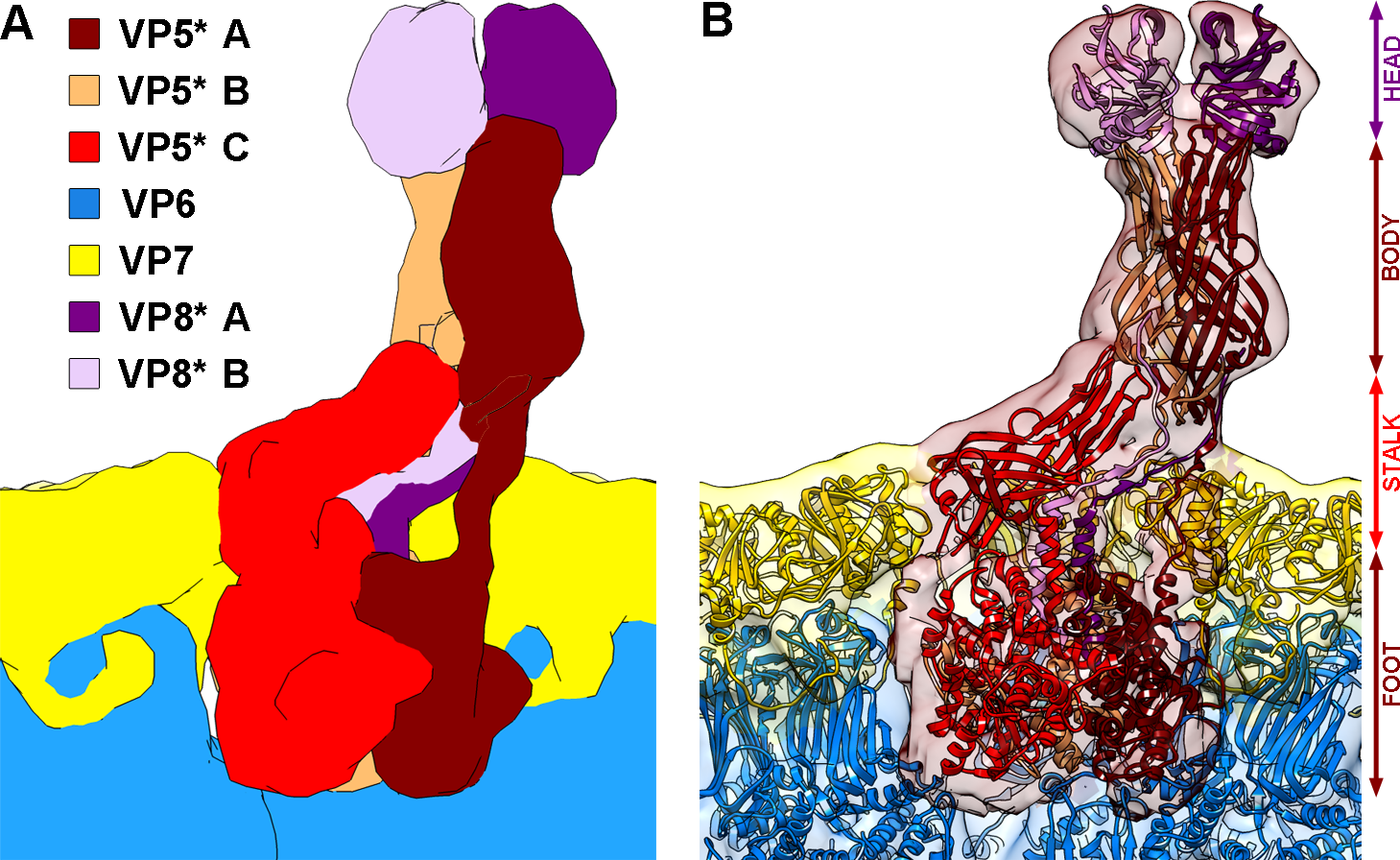

Supplement: Figure S3 — Fit of the atomic coordinates into the cryo-EM map. (A) Scheme of the NTR spike and its interaction with VP6 and VP7 shells. Proteins and color coding are indicated. (B) Superposition of the atomic coordinates of RRV TR-TLP (PDBs 3N09 and 2GH8) [6] and the SA11 NTR-TLP cryo-EM map. Coordinates and densities are color-coded as in A. (TIF) [file ppat.1004157.s003.tif]

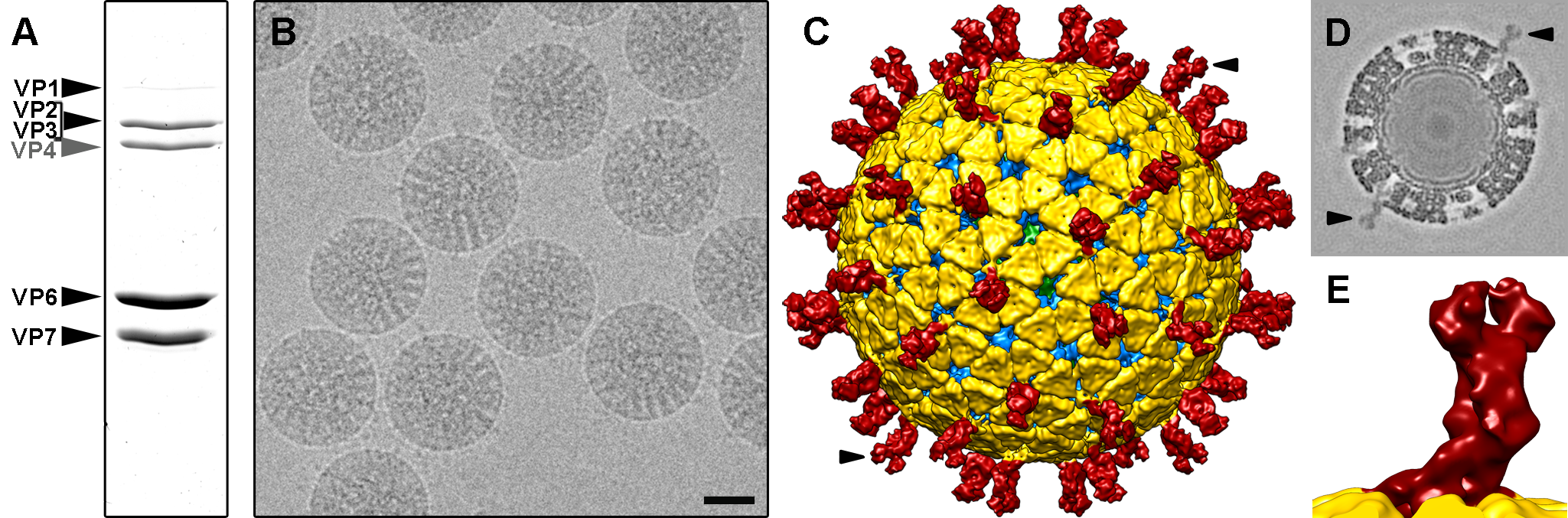

Supplement: Figure S4 — Biochemical and structural analysis of SA11 NTR-TLP grown in the absence of leupeptin. (A) Coomassie blue-stained SDS-PAGE gels of purified SA11 TLP grown in the absence of trypsin and leupeptin. Positions of structural viral proteins (VP) are indicated. Position of unprocessed spike protein VP4 is highlighted in grey. (B) Cryo-electron micrograph of vitrified particles. The bar represents 50 nm. (C) Surface-shaded representation of the outer surface of the 3DR, viewed along an icosahedral 2-fold axis. The surface is radially color-coded to represent VP4 (red), VP7 (yellow) and VP6 (blue). The density is contoured at 1 σ above the mean. (D) Transverse sections, 2.8 Å thick, taken from the maps parallel but displaced 34 Å from the central section, viewed along a 2-fold axis (darker, denser). Arrows indicate spikes in the surface-shaded representations in C and their corresponding densities in D. (E) Close-up view of the spike represented as in C. (TIF) [file ppat.1004157.s004.tif]

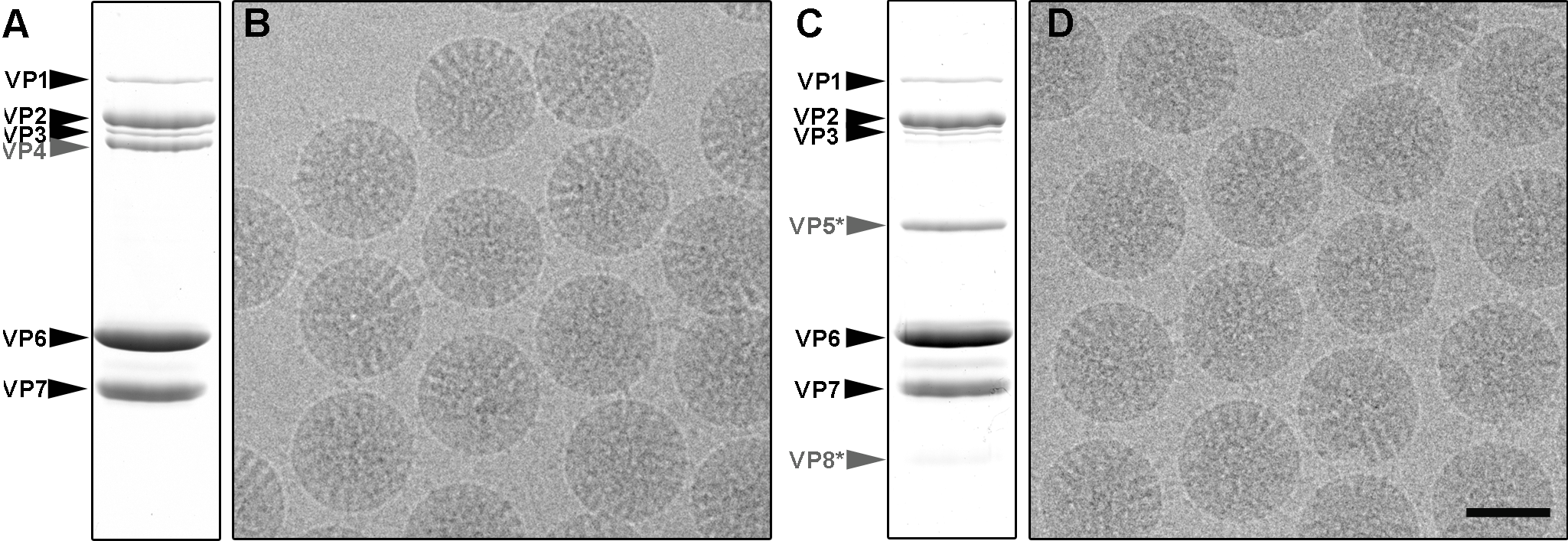

Supplement: Figure S5 — Analysis of OSU NTR- and TR-TLP by SDS-PAGE and cryo-EM. (A, C) Coomassie blue-stained SDS-PAGE gels of purified OSU TLP grown in the absence (A) or presence (C) of trypsin. Positions of structural viral proteins (VP) are indicated. Unprocessed spike protein VP4 and its proteolytic products VP8* and VP5* are highlighted in grey. (B, D) Cryo-electron micrographs of NTR (B) and TR (D) particles. The bar represents 50 nm. (TIF) [file ppat.1004157.s005.tif]

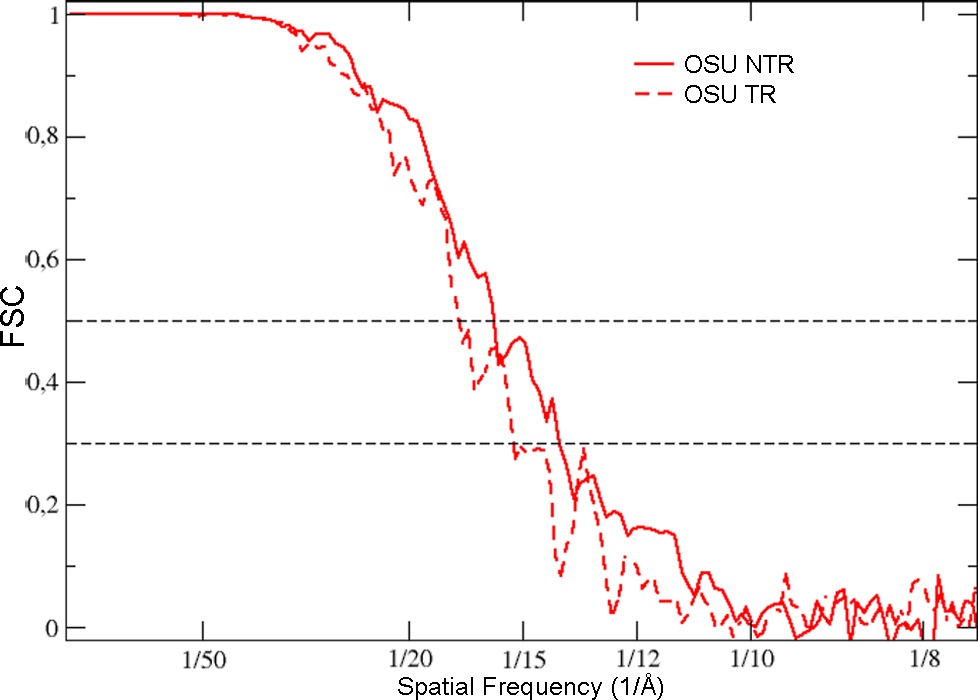

Supplement: Figure S6 — Assessment of the resolution cryo-EM 3DR of OSU NTR and TR TLP. FSC resolution curves were calculated for OSU NTR-TLP (red, continuous line) and TR-TLP (red, dashed line). For the 0.5 threshold, values for OSU NTR- and TR-TLP were 16.2 and 17.4 Å, respectively; values for the 0.3 threshold were 14.3 and 15.4 Å, respectively. (TIF) [file ppat.1004157.s006.tif]

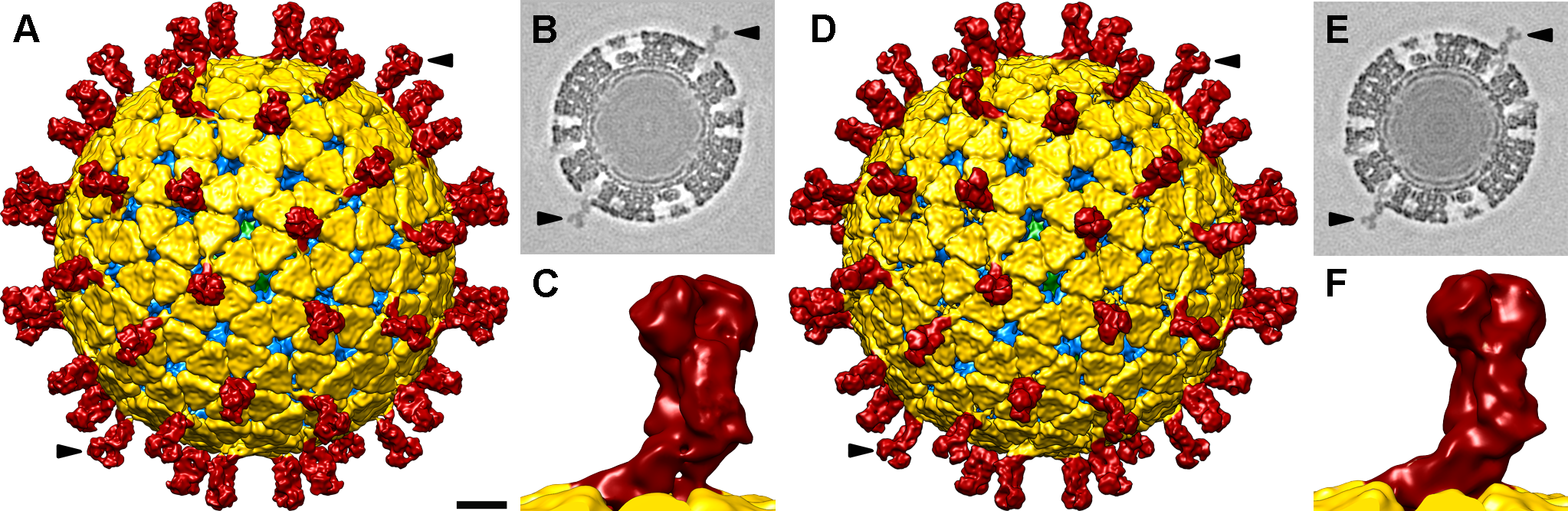

Supplement: Figure S7 — Single-particle three-dimensional structures of OSU NTR- and TR-TLP. (A, D) Surface-shaded representation of the outer surfaces of NTR (A) and TR (D) particles, viewed along an icosahedral 2-fold axis. The surfaces are radially color-coded to represent VP4 or VP8*/VP5* spikes (red), VP7 (yellow) and VP6 (blue). The density is contoured at 1σ above the mean. The bar represents 100 Å. (B, E) Transverse sections, 2.8 Å thick, taken from the maps of NTR (B) and TR (E) TLP, parallel but displaced 34 Å from the central section, viewed along a 2-fold axis (darker, denser). Arrows indicate spikes in the surface-shaded representations in C and D and their corresponding densities in B and E. (C, F) Close up view of the NTR (C) and TR (F) spike represented as in A and D. (TIF) [file ppat.1004157.s007.tif]
