# Supplementary material for: New Insights into Rotavirus Entry Machinery: Stabilization of Rotavirus Spike Conformation Is Independent of Trypsin Cleavage
Source: PLoS Pathog. 2014 May 29;10(5):e1004157. doi: 10.1371/journal.ppat.1004157 (PMC4038622; doi:10.1371/journal.ppat.1004157)
Supplement: Table S1 — GenBank accession numbers for SA-C4111 and OSU-C5111 genomic segments. (DOC) [file ppat.1004157.s008.doc]

**Table S1. GenBank accession numbers** for SA-C4111 and OSU-C5111 genomic segments

| Strain | Segment | Accession number |
| --- | --- | --- |
|  | 1 | KJ450831 |
|  | 2 | KJ450832 |
|  | 3 | KJ450833 |
|  | 4 | KJ450834 |
|  | 5 | KJ450835 |
| SA-C4111 | 6 | KJ450836 |
|  | 7 | KJ450837 |
|  | 8 | KJ450838 |
|  | 9 | KJ450839 |
|  | 10 | KJ450840 |
|  | 11 | KJ450841 |
|  | 1 | KJ450842 |
|  | 2 | KJ450843 |
|  | 3 | KJ450844 |
|  | 4 | KJ450845 |
|  | 5 | KJ450846 |
| OSU-C5111 | 6 | KJ450847 |
|  | 7 | KJ450848 |
|  | 8 | KJ450849 |
|  | 9 | KJ450850 |
|  | 10 | KJ450851 |
|  | 11 | KJ450852 |
