# Supplementary material for: New Insights into Rotavirus Entry Machinery: Stabilization of Rotavirus Spike Conformation Is Independent of Trypsin Cleavage
Source: PLoS Pathog. 2014 May 29;10(5):e1004157. doi: 10.1371/journal.ppat.1004157 (PMC4038622; doi:10.1371/journal.ppat.1004157)
Supplement: Table S2 — EMDB accession codes for density maps. (DOC) [file ppat.1004157.s009.doc]

**Table S2. EMDB a**ccession codes for density maps

| Strain | Accession code |
| --- | --- |
| SA11 NTR-TLP | EMD-2573 |
| SA11 TR-TLP | EMD-2574 |
| SA NTR-TLP -leupeptin | EMD-2575 |
| OSU NTR-TLP | EMD-2576 |
| OSU TR-TLP | EMD-2577 |
| Class 1 NTR spike | EMD-2578 |
| Class 2 NTR spike | EMD-2579 |
| Class 1 TR spike | EMD-2580 |
