# Supplementary material for: New Insights into Rotavirus Entry Machinery: Stabilization of Rotavirus Spike Conformation Is Independent of Trypsin Cleavage
Source: PLoS Pathog. 2014 May 29;10(5):e1004157. doi: 10.1371/journal.ppat.1004157 (PMC4038622; doi:10.1371/journal.ppat.1004157)
Supplement: Text S1 — Supporting Materials and Methods. (DOC) [file ppat.1004157.s010.doc]

**Supporting Materials and Methods**

**Production and purification of rotavirus TLP*.*** TLP were prepared by infecting 3-day post-confluent monolayers of MA104 cells with a multiplicity of infection of 25 PFU/cell. Viruses were activated by digestion with 100 BAEE U/ml of TPCK-treated trypsin (17,768 BAEE U/mg of protein; TPCK Trypsin, Thermo Scientific Pierce; 30 min, 37ºC). Prior to adsorption, MA104 monolayers were washed twice with MEM medium and the virus inoculum was added and allowed to adsorb (90 min, 37ºC). Cell monolayers were then washed twice with MEM. For the preparation of TLP in the presence of trypsin (trypsinized rotavirus; TR), MEM containing 10 BAEE U/ml TPCK-trypsin was added. For the preparation of rotavirus TLP with intact VP4 (non-trypsinized rotavirus, NTR), cell monolayers were washed three times with MEM after virus adsorption and MEM containing 0.5 μg of the protease inhibitor leupeptin was added. Cells and extracellular media were harvested 24 h post-infection (hpi) and separated by centrifugation (12,000xg, 30 min, 4ºC). The supernatant was adjusted to 10% PEG-8000 and 2.3% NaCl, incubated (overnight, 4ºC) with gentle agitation, centrifuged (10000xg, 30 min, 4ºC), and the pelleted virus was resuspended in a small volume of TBS (25 mM Tris base, 0.7 mM Na_2_PO_4_, 5.6 mM glucose, 136.9 mM NaCl, 5.1 mM KCl, 1 mM MgCl_2_, 1 mM CaCl_2_) [1]. The pellet of infected cells was resuspended in a small volume of medium, frozen and thawed three times to release cell-associated virus, and clarified by centrifugation (12,000xg, 30 min, 4ºC). The virus-containing supernatant was pooled with the PEG-8000-concentrated virus and semi-purified through a cushion of 40% sucrose in TBS by centrifugation (70,000xg, 2.5 h, 4ºC). The pellet was resuspended in TBS and extracted twice with 1,1,1,2,3,4,4,5,5,5-decafluoropentane (Sigma-Aldrich). Highly purified TLP were isolated from this extract by double band isolation in CsCl gradients as described by Patton et al. [1]. Purified TLP were dialyzed extensively against TBS and stored at 4ºC. TR- and NTR-TLP were also purified following the procedure described by Crawford et al. [2], without the use of protein inhibitors for purification of NTR-TLP. Samples were resolved by SDS-PAGE and stained with Coomassie blue or analyzed by western blot with an anti-VP4 antibody which recognizes VP5* and the precursor VP4, (a kind gift from Dr. C. Eichwald, Virologisches Institut, Universität Zürich, Switzerland). The amount of TLP in the preparations was determined by densitometric analysis of Coomassie stained SDS-PAGE gels, assuming that VP6 correspond to 37% of total viral protein mass.

**Determination of viral infectivity.** Viral infectivity was determined by plaque assays and fluorescent focus assays essentially as described by Arnold et al. [3]. NTR and TR-TLP were treated (30 min, 37ºC) with 0 or 100 U/ml TPCK-trypsin. A sample of activated and mock-activated virus was treated immediately with Laemmli sample buffer (5 min, 100ºC), resolved in a 12% SDS-PAGE gel and Coomassie-stained.

For plaque assays, triplicate samples of viruses activated with 100 U/ml TPCK-trypsin were serially diluted and adsorbed to confluent MA104 monolayers (90 min, 37ºC); monolayers were washed and overlaid with 0.6% noble agar in MEM medium with 10 U/ml TPCK-trypsin. Plaques were fixed at 4-5 days after infection with 4% formaldehyde and stained with crystal violet.

For fluorescent focus assays, triplicate samples of mock-activated viruses were serially diluted and adsorbed to confluent MA104 monolayers (90 min, 37ºC). Following virus adsorption, the inoculum was removed and monolayers washed three times with MEM before addition of MEM with 2% fetal calf serum. Infection was allowed to continue for 16 h, after which infected cells were washed with cold PBS and methanol-fixed. Infected cells were detected by incubation with a 1:500 dilution of rabbit antiserum to a purified protein containing the 140 C-terminal amino acids of the SA11-C4111 NSP4 protein fused to a 6xHis tag (Davids Biotechnology, Regensburg, Germany). Bound antibodies were detected by incubation with Alexa568-conjugated goat anti-rabbit secondary antibody (1:500; Life Technologies). Cells were imagined with a Leica SP5 confocal microscope. Images were analyzed using Fiji software [4]. Statistical analysis was performed using Student's t-test for unpaired data. Data are expressed as mean ± SD.

**Supporting References**

1. Patton JT, Chizhikov V, Taraporewala Z, Chen D (2000) Virus replication. Methods Mol Med 34: 33-66.

2. Crawford SE, Mukherjee SK, Estes MK, Lawton JA, Shaw AL, et al. (2001) Trypsin cleavage stabilizes the rotavirus VP4 spike. J Virol 75: 6052-6061.

3. Arnold M, Patton JT, McDonald SM (2009) Culturing, storage, and quantification of rotaviruses. Curr Protoc Microbiol Chapter 15: Unit 15C 13.

4. Schindelin J, Arganda-Carreras I, Frise E, Kaynig V, Longair M, et al. (2012) Fiji: an open-source platform for biological-image analysis. Nat Methods 9: 676-682.
